# Helleborein

**Published:** 1882-02

**Authors:** 


### HELLEBOREIN.—A NEW HEART STIMULANT.

Helleborein has been used sufficiently in the New York Hospital to warrant some conclusions concerning its employment, It is an active principle common to the Helleborus Viridis and Helleborus Niger, the alkaloid obtained from the former being, however, much stronger than that obtained from the latter. In appearance Helleborein is a light yellow amorphous powder, freely soluble in water or alcohol. The watery solution has been preferred, and this is used hypodermically as the local action of the drug upon the stomach and intestine is found to be irritant. This local action, produced by the drug given in a solid form or in concentrated solution, has been known for some time, and it has been usually classed among the drastic cathartics on account of its very irritant action upon the gastro-intestinal tract. Its constitutional effects have heretofore escaped notice either from the fact that they were obscured by the local action, or from the fact that the drug was so rapidly eliminated by the intestine that too little was absorbed to produce any constitutional disturbance. It is known that its administration by the stomach delays its absorption, and is not followed at once by general effects. The method of administration most favorable to the production of its constitutional effects, is its introduction directly into the circulation by injection into a vein or what is almost as effective, by its injection under the skin. It is not found to act as an irritant on the cellular tissue, and has not been followed by any local disturbance more serious than temporary hyperæmia.

Upon the heart the action of the drug is very marked. It increases the force and diminishes the frequency of the heart's action, acting in a manner very similar to that of Digitalis. This effect is best produced by small doses repeated. In a number of cases of cardiac disease this action has fully been demonstrated. Where the heart action was feeble, rapid and irregular owing to the dilatation following valvular lesions, the benefit derived from the use of Helleborein was very apparent. Doses of one-fifteenth grain, hypodermically, repeated every two hours, produced a decided modification in the rapidity and irregularity, and increased the power of the heart's action to the immediate relief of the urgent symptoms of cyanosis and dyspnœa. This effect was continued until the heart became regular and forcible and the distressing symptoms subsided. In cases of irregular action dependent on nervous excitement, or of palpitation attended with dyspnœa, where no valvular lesion could be made out, the action of the drug was equally apparent. After two or three doses the change in the rhythm of the beats was marked, and the pulse became regular, slower and as it recovered its tone the symptoms of discomfort were relieved. In these cases the action of the drug in increasing the arterial tension was very noticeable.

In cases of heart failure, complicating pneumonia, typhoid fever and other conditions attended by rise of temperature, Helleborein has been used successfully as a cardiac stimulant. In one case of pneumonia with complicatory œdema of the lungs due to failure of the heart the drug was employed with marked effect. The patient was moribund, all efforts directed toward stimulation of the heart and relief of the œdema having proved failures. Hypodermics of Helleborein were first tried after the radial pulse had ceased. Two minutes after the injection of one-tenth of a grain, the radial pulse returned and the heart action was maintained for two hours by the use of the drug. In this case the action as a respiratory, as well as a cardiac stimulant was marked, and had the drug been employed earlier it is possible that the cardiac failure might have been overcome. The advantages of Helleborein over Digitalis are numerous. If given hypodermically it never produces nausea or vomiting. Its effects are not cumulative and no unfavorable results followed when it was continued for several weeks steadily. There seems to be no danger of heart failure following undue exertion where it has been given some time, as occasionally is seen where Digitalis has been used. It does not produce the disagreeable cerebral symptoms which often follow the use of Digitalis. In several cardiac cases it was used with good effect after Digitalis had been tried but had produced no amelioration of the symptoms. Its action upon the kidneys is less marked than that of Digitalis. It produces a diuretic action probably by increasing the arterial tension and by regulating the heart and thus increasing the blood pressure. Thus, in cases of ascites due to cardiac disease it fulfils two modifications, by stimulating the action of the kidneys as well as regulating the action of the heart. But as a diuretic it is inferior to Digitalis. A careful experimental study of the physiological action of the drug on the various organs has not been made, but sufficient is already known to show that in Helleborein we have an efficient heart stimulant and diuretic.—*Chicago Medical Review.*

